# Added diagnostic value of 16S rRNA gene pan-mycobacterial PCR for nontuberculous mycobacterial infections: a 10-year retrospective study

**DOI:** 10.1007/s10096-019-03621-z

**Published:** 2019-07-16

**Authors:** Simon Andenmatten, Onya Opota, Jessica Mazza-Stalder, Laurent Nicod, Gilbert Greub, Katia Jaton

**Affiliations:** 1 Institute of Microbiology, University of Lausanne and Lausanne University Hospital, Lausanne, Switzerland; 2 Division of Pulmonology, University of Lausanne and University Hospital of Lausanne, Lausanne, Switzerland

**Keywords:** Mycobacteria, Nontuberculous mycobacteria, Infection, Pulmonary infection, Extra-pulmonary infection, Pan-mycobacterial PCR, Molecular diagnostics, Microscopy, Acid-fast bacilli, Auramine staining, Mycobacterial culture, Polymerase chain reaction, 16S rRNA gene

## Abstract

The diagnosis of mycobacterial infections has been dramatically improved by the introduction of molecular methods aimed to reduce the time to diagnosis as compared with culture. The broad range pan-mycobacterial PCR can detect all the mycobacterial species directly from clinical specimens. We aimed to evaluate its usefulness and its clinical added value for the diagnosis of nontuberculous mycobacterial (NTM) infections. We performed a retrospective study (2003–2013) including 952 samples taken from 639 patients with clinical suspicion of NTM infection. The performance of smear microscopy, PCR and culture was established using clinical data to investigate discrepant results. We also compared the time to microbial diagnosis between the direct PCR and culture. The sensitivity, specificity, positive and negative predictive values of the PCR were 61.6% (53.5–69.1), 99.1% (98.2–99.6), 92.8% (85.8–96.5) and 93.4% (91.6–94.9), respectively, when considering all specimens. When considering smear-positive specimens and smear-negative specimens, the sensitivity was 81.6% and 40%, respectively. The sensitivity for pulmonary and extra-pulmonary smear-positive specimens was 85.2% versus 72.7%. The median time to identification at species level was 35 days (SD, 17.67) for culture and 6 days (SD, 2.67) for the PCR (when positive), which represents a 29-day shorter time to results (*p* < 0.0001). The 16S rRNA gene pan-mycobacterial PCR displays a substantial benefit in terms of time to diagnose NTM infections when compared with culture. Despite an excellent specificity, its sensitivity is yet limited in particular for smear-negative specimens, which might be improved by relying onto real-time PCRs.

**Electronic supplementary material:**

The online version of this article (10.1007/s10096-019-03621-z) contains supplementary material, which is available to authorized users.

## Introduction

Nontuberculous mycobacteria (NTM), in contrast to *Mycobacterium tuberculosis* complex species, are bacteria widely spread in the environment and can be found in a broad range of ecosystems such as soils and water, including drinking water systems [1–3]. NTM are opportunistic pathogens associated with both pulmonary and extra-pulmonary infections depending on the species. Their medical importance has recently raised due to the increasing number of immunocompromised hosts (solid organ transplant recipients and oncologic patients among others) and due to the modern tools that improved their detection in clinical sample [1, 2, 4]. While most of the NTM are non-pathogenic, the pathogenic NTM are generally causing pulmonary infections (90% of cases) whereas extra-pulmonary manifestations can involve any organ and tissues, causing for instance lymphadenitis, skin infections, bones or soft tissue or may be disseminated [1, 2, 4, 5].

While clinical manifestations of NTM vary according to the species and to the route of infection (inhalation, ingestion or inoculation), different species can lead to similar diseases but may require distinct treatments, which makes the microbiological diagnosis important [1, 2, 4]. Due to their structural characteristics, mycobacteria are naturally resistant to many antibiotics and treating NTM infections requires a prolonged treatment with a combination of multiple antimicrobial molecules, which choice is based on the species identification and on the results of antibiotic susceptibility testing [3, 5].

Smear-examination of clinical samples to detect acid-fast bacilli (AFB) was historically the first microbiological test for the diagnosis of mycobacterial infection. However, this method has a limited sensitivity and specificity and does not provide any hint on the mycobacterial species. In particular, smear microscopy cannot distinguish mycobacteria of the complex *tuberculosis* from NTM [6]. So far, culture represents a reference method for microbiological diagnosis of mycobacteria due to a low limit of detection (10 to 100 viable organisms per millilitre) [7]. Culture also provides a pure isolate for subsequent antimicrobial susceptibility testing. However, culture is affected by the slow growth of mycobacteria especially for the so-called slow-growing species as they need more than 7 days to form a colony (as compared with about 48–72 h for rapidly growing species). In addition, infections due to some mycobacteria may not be detected by conventional culture because of the requirement of specific nutrients such as hemin for *Mycobacterium haemophilum* or specific temperature culture conditions of 30 °C for *Mycobacterium marinum* and of 42 °C for *Mycobacterium xenopi*. Finally, some mycobacteria such as *Mycobacterium leprae* remain uncultivable in vitro.

During the last decades, several molecular methods have been developed for the detection and identification of *M. tuberculosis* complex and NTM directly from clinical samples, in order to circumvent the slow or difficult growth of these organisms. Molecular methods have the potential to shorten the diagnosis from several weeks to days or even hours. The aim of this study was to evaluate the usefulness of a pan-mycobacterial PCR targeting the 16S rRNA–encoding gene. This PCR has the potential to detect any mycobacterial species and when positive to provide further identification at species (or complex) level by Sanger sequencing of the obtained amplicon. We conducted a retrospective study analysing the results of cultures, smear microscopy and 16S rRNA gene PCR, over a 10-year period representing samples taken from patients suspected to suffer from pulmonary or extra-pulmonary infection due to nontuberculous mycobacteria.

## Method

### Study design

**Fig. 1:**
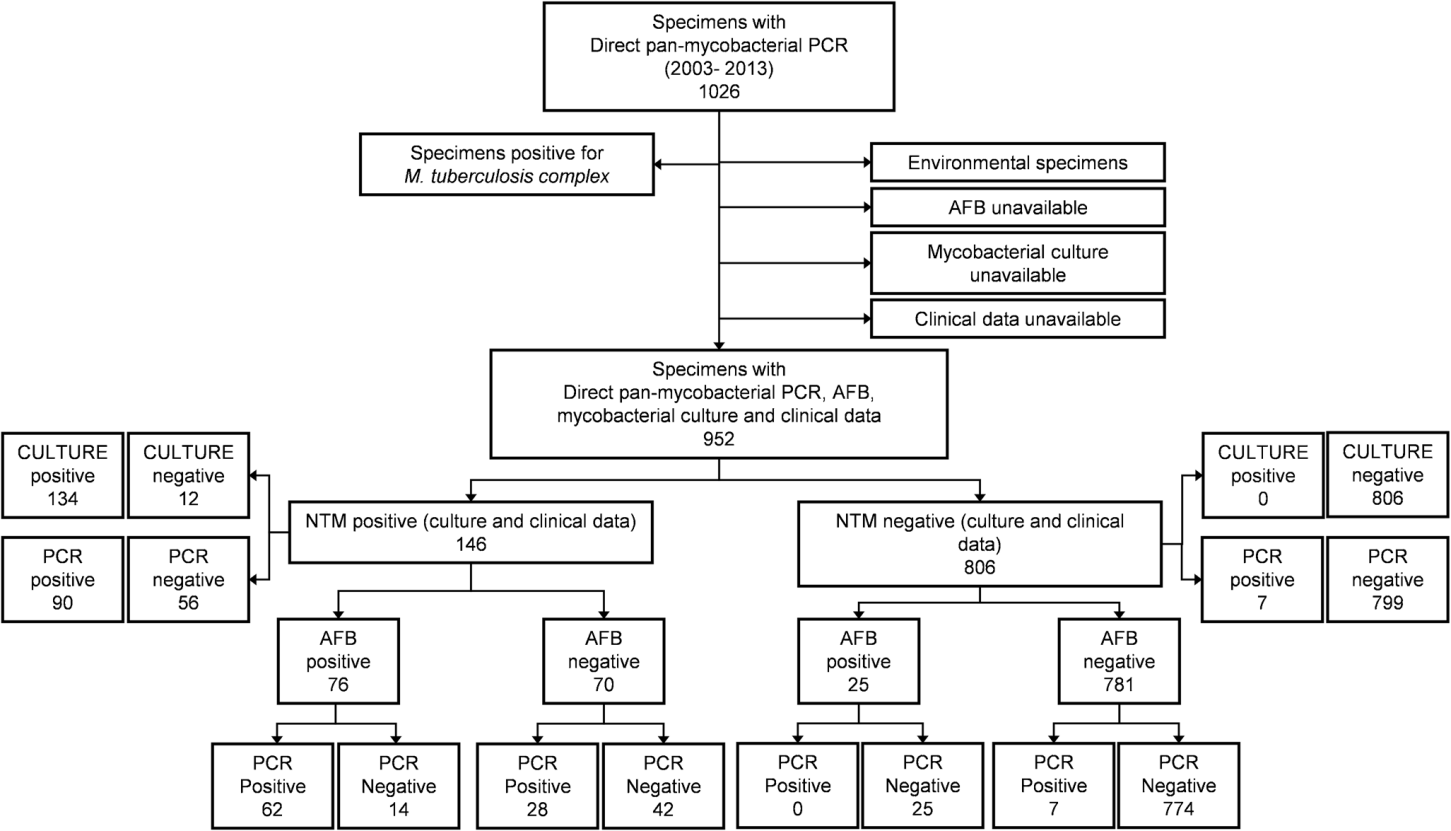
Study design and specimens distribution. The diagnostic performance of the different tests was established using clinical data to investigate discrepant results

The retrospective study was conducted over a period of 10 years (2003–2013) for which a mycobacterial infection was suspected and specimens were analysed in the Laboratory of Molecular Diagnostic and Mycobacteria of the Institute of Microbiology of the Lausanne University Hospital, a 1000-bed tertiary-care hospital located in Lausanne, Switzerland. Using the laboratory information system (LIS) of our hospital, we achieved a comprehensive extraction of all analyses corresponding to mycobacterial diagnostic requests between 2003 and 2013. Samples positive for *Mycobacterium tuberculosis* complex and patients for which clinical data were not available were excluded. This resulted to the inclusion of 952 specimens corresponding to 639 patients (Fig. 1).

We performed an analysis (i) “per sample” to establish the analytical performance (sensitivity, specificity, positive and negative predictive values) of the pan-mycobacterial PCR, considering culture and clinical data as the gold standard and (ii) “per patient” to establish the time to microbiological diagnostic for the pan-mycobacterial PCR and culture. To assess the performance of the direct pan-mycobacterial PCR, we used first culture, then clinical data as reference, an approach that we previously applied to determine the performance of *M. tuberculosis* rapid molecular tests (Table S1) [6, 8]. In particular, specimens with positive PCR and negative culture were considered true positive (i) if the same specimen had a positive smear microscopy; (ii) if another specimen of the same patient had a positive PCR; (iii) if the identified NTM was a fastidious or uncultivable microorganism, namely *M. genavense*, *M. marinum*, *M. ulcerans*, *M. haemophilum* or *M. leprae*; and (iv) if based on the clinical data. For the second analysis “per patient”, only patients with a positive NTM culture for which the results of direct smear microscopy and pan-mycobacterial PCR were available were included. For a given patient, repeated infections with the same mycobacterial species were considered independently and included again if more than 12 months separated both positive PCRs. In addition, when multiple samples gave a positive result for a given infection episode, the fastest microbiological result was considered to calculate the time to result.

### Ethical issues

The study was approved by the local ethics committee (Commission Cantonale d’Ethique de la Recherche sur l’Etre Humain, Lausanne, Switzerland), protocol 372/13.

## Microbiology methods

Smear examination for acid-fast bacilli detection was performed by staining heat-fixed samples with a fluorescent auramine-thiazine red [6, 8, 9]. Smear grading was determined according to the International Union Against Tuberculosis and Lung Disease scale. Solubilisation of purulent samples was achieved using the mucolytic agent N-acetyl-l-cysteine (2% *m*/*v* pH 6.8). For any positive direct examination for AFB, a *Mycobacterium tuberculosis*–specific PCR was performed without waiting for the result of the culture [10]. In case of negative *M. tuberculosis*–specific PCR, a pan-mycobacterial PCR was performed.

Mycobacterial cultures were achieved in Mycobacteria Growth Indicator Tube (Becton Dickinson, Heidelberg, Germany) after sample treatment with NaOH in order to eliminate bacteria that constitute the flora of non-sterile samples and incubated for up to 8 weeks in the automated growth detection system BACTEC MGIT 960 (Beckton Dickinson) [9]. Culture conditions were adjusted when infection with *M. haemophilum* (culture supplemented with hemin), *M. marinum* (grows temperature of 30 °C) or *M. xenopi* (grows temperature at 42 °C) were suspected. The presence of mycobacteria in positive culture was determined by AFB detection using a Ziehl-Neelsen staining; if positive, the presence/absence of *M. tuberculosis* complex was evaluated using the *M. tuberculosis* complex specific antigen test (BD MGIT TBc Identification Test, Beckton Dickinson). When negative for *M. tuberculosis* complex, the mycobacterial strain was identified using pan-mycobacterial 16S rRNA gene PCR and, when necessary, pan-mycobacterial *rpoB* PCR as well as pan-mycobacterial *hsp65* PCR as described below.

### Pan-mycobacterial 16S rRNA gene PCR

The pan-mycobacterial PCR targets the 16S rRNA gene with forward primers 5′-TGCACACAGGCCACAAGGGA-3′ and reverse primers 5′-GAGAGTTTGATCCTGGCTCAG-3′ specific for the genus *Mycobacterium* as previously reported [10, 11]. During the studied period, a nested PCR was carried to increase the sensitivity of this method used directly on clinical specimens. It consisted in using a second pair of primers nested (NF 5′-CTTAACACATGCAAGTCGAAC-3′ and NR 5′-TTTCACGAACAACGCGACAA-3′) within the first amplification product. This significantly improves sensitivity because of the double amplification, but the risk of contamination is higher. The product of this double amplification was then sequenced with primer 5′-CCCACTGCTGCCTCCCGTAG-3′ and primer 5′-CTTAACACATGCAAGTCGAAC-3′, and the obtained nucleotide signature sequence is compared with other signature sequences, which allows the determination of the name of the mycobacterial species that was amplified [11, 12].

### Data analysis and statistics

The databases were analysed with the Stata software (Stata Statistical Software 2011, Release 12, Stata Corporation, College Station, TX). Median times to results were compared using the Wilcoxon-Mann-Whitney test. A Student *t* test was used to determine the independent and non-equal variances. A *p* value < 0.05 was considered statistically significant.

## Results

### Patients and samples

**Fig. 2:**
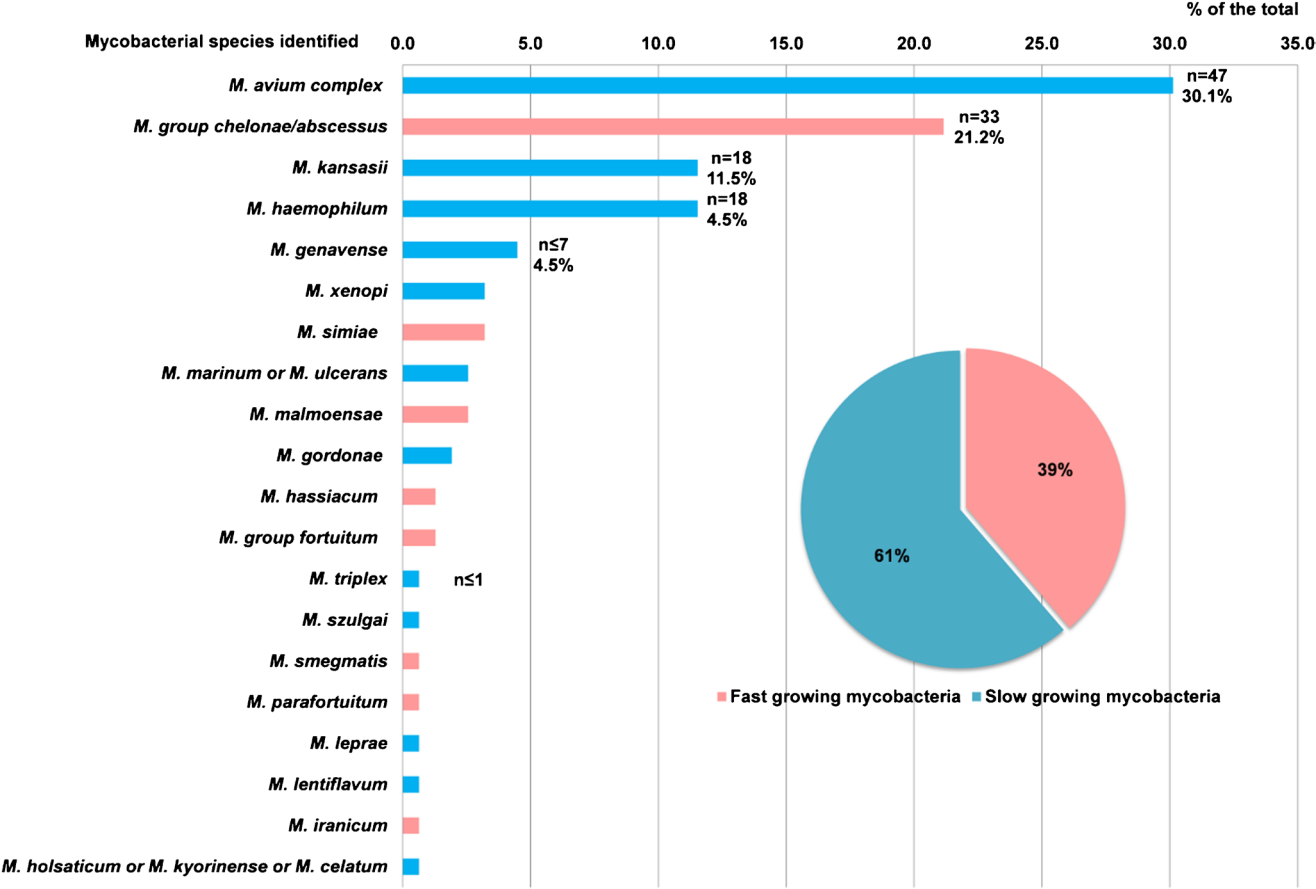
Mycobacterial species identified

Our study included 952 samples collected between 2003 and 2013 (corresponding to 639 patients) with a direct smear examination, a mycobacterial culture, and a direct 16S rRNA gene pan-mycobacterial PCR. Three environmental specimens were excluded (Fig. 1). Among the 952 specimens, 15.3% (*n* = 146) had a positive culture and 84.6% (*n* = 806) had a negative culture (Fig. 1 and Table 1). A total of 97 specimens (10.2%) had positive direct pan-mycobacterial PCR. Among all the species identified, either by culture or direct pan-mycobacterial PCR, 61% were slow-growing or 39% were fast-growing mycobacteria (Fig. 2). The top five mycobacterial species identified were *M. avium* complex (30.1%), *M.* group *abscessus-chelonae* (21.2%), *M. kansasii* (11.5%), *M. haemophilum* (11.5%) and *M. genavense* (4.5%). During the studied period, an outbreak of *M. haemophilum* occurred, which explains that this organism is found in the top five (Fig. 2) [11].

**Table 1:** Performance of the smear microscopy on a total of 952 samples. *PPV* positive predictive value, *NPV* negative predictive value

	Sensitivity % (95% CI)	Specificity % (95% CI)	PPV % (95% CI)	NPV % (95% CI)
All specimens (952)	52.1 (44.0–60.0) (77/146)	96.9 (95.5–97.9) (780/806)	75.2 (66.0–82.6) (77/102)	91.8 (89.7-93.4) (780/850)
Pulmonary (347)	62.1 (51.6–71.5) (54/87)	95 (90.7–97.1) (247/260)	80.6 (70.6–88.3) (54/67)	88.2 (83.9–91.5) (247/280)
Extra-pulmonary (605)	38.3 (271–51.0) (23/60)	97.8 (96.2–98.7) (533/545)	65.7 (49.1–79.2) (23/35)	93.5 (91.2–95.2) (533/570)

## Sensitivity, specificity and predictive value of smear microscopy

Smear microscopy, which is historically the first microbiological test, performed for the diagnosis of tuberculosis may vary a lot in terms of sensitivity, specificity and predictive values according to the region, to the prevalence of mycobacterial infections and to the experimenter. We established the performance of smear microscopy on the 952 clinical specimens using clinical data to investigate discrepant results between smear microscopy, pan-mycobacterial PCR and mycobacterial culture (Table 1 and Table S1). The sensitivity, specificity, PPV and NVP of the smear microscopy were 52.1% (77/146), 96.9% (780/806), 75.2% (77/102) and 91.8% (780/850). When considering pulmonary specimens the sensitivity, specificity, PPV and NVP of the smear microscopy were 62.1% (54/87), 95% (247/260), 80.6% (54/67) and 88.2% (247/280). When considering extra-pulmonary specimens, the sensitivity, specificity, PPV and NVP were 38.3% (23/60), 97.8% (533/545), 65.7% (23/35) and 93.5% (533/570). The data suggest a limited sensitivity and low PPV of the smear microscopy for the detection of NTM especially for extra-pulmonary specimens. The specificity and the NPV of the smear microscopy is good. However, the NPV might have been artificially increased by the low prevalence of NTM infections.

### Sensitivity, specificity and predictive value of mycobacterial culture

To address the diagnostic performance of culture, we used both microbiological findings and clinical data as reference (Table 2 and Table S1). When considering all specimens, the sensitivity, specificity, PPV and NPV of culture were 91.8% (134/146), 100% (806/806), 100% (134/314) and 98.5% (806/818). When considering only pulmonary specimens, the sensitivity, specificity, PPV and NPV of culture were 98.8% (86/87), 100% (260/260), 100% (86/86) and 99.6% (260/261). When considering extra-pulmonary specimens, the sensitivity, specificity, PPV and NPV of culture were 81.4% (48/59), 100% (546/546), 100% (48/48) and 98% (546/557). Among PCR-positive culture, negative specimens were *M. leprae* (*n* = 1), *M. genavense* (*n* = 4) and *M. marinum*/*ulcerans* (*n* = 3).

**Table 2:** Performance of culture on a total of 952 samples. *PPV* positive predictive value, *NPV* negative predictive value

	Sensitivity % (95% CI)	Specificity % (95% CI)	PPV % (95% CI)	NPV % (95% CI)
All specimens (952)	91.8 (86.2–95.2) (134/146)	100 (99.5–100) (806/806)	100 (97.2–100) (134/134)	98.5 (97.2–100) (806–818)
Pulmonary (347)	98.8 (93.7–99.9) (86/87)	100 (98.5–100) (260/260)	100 (95.7–100) (86/86)	99.6 (97.7–99.9) (260/261)
Extra-pulmonary (605)	81.4 (69.6–89.3) (48/59)	100 (99.3–100) (546/546)	100 (92.6–100) (48/48)	98.0 (96.5–98.9) (546/557)

### Sensitivity, specificity and predictive value of the pan-mycobacterial PCR

The global performance of the direct pan-mycobacterial PCR for the detection of NTMs was achieved using first culture as reference then using microbiological and clinical data as reference. When considering all the 952 clinical specimens, including pulmonary and extra-pulmonary specimens as well as smear-positive and smear-negative specimens and using clinical data to investigate microbiological discrepant results, the direct pan-mycobacterial PCR exhibited a sensitivity of 61.6% (90/146), a specificity of 99.1% (799/806), a positive predictive value (PPV) of 92.8% (90/97) and a negative predictive value of 93.4% (799/855) (Table 3 and Table S1). When considering only smear-positive specimens, the sensitivity, specificity, PPV and NPV were 81.6% (62/76), 100% (25/25), 100% (62/62) and 64.1% (25/39). When considering only smear-negative specimens, the sensitivity, specificity, PPV and NPV were 40% (29.3–51.7), 99.1% (98.2–99.6), 80% (64.1–90.0) and 94.8% (93.1–96.2) (Table 4).

**Table 3:** Performance of the direct 16S rRNA gene pan-mycobacterial PCR. The global performance of the direct 16S rRNA gene pan-mycobacterial PCR was calculated first using culture as reference (culture) then using clinical data to investigate discrepant results between smear microscopy, PCR and culture (culture and clinical data). Total of 952 samples. *PPV* positive predictive value, *NPV* negative predictive value

	Reference	Sensitivity % (95% CI)	Specificity % (95% CI)	PPV % (95% CI)	NPV % (95% CI)
All specimens (953)	Culture	58.2 (49.7–66.2) (78/134)	97.7 (96.3–98.4) (799/818)	80.4 (71.4–87.1) (78/97)	93.4 (91.6–94.9) (799/855)
	Culture and clinical data	61.6 (53.5–69.1) (90/146)	99.1 (98.2–99.6) (799/806)	92.8 (85.8–96.5) (90/97)	93.4 (91.6–94.9) (799/855)
All smear-positive specimens (102)	Culture	80.3 (69.6–87.8) (57/71)	100 (86.7–100) (25/25)	100 (93.7.100) (57/57)	64.1 (48.4–77.3) (25/39)
	Culture and clinical data	81.6 (71.4–88.7) (62/76)	100 (86.7–100) (25/25)	100 (94.2.100) (62/62)	64.1 (48.4–77.3) (25/39)
All smear-negative specimens (851)	Culture	35.4 (24.9–47.5) (23/65)	99.1 (98.2–99.6) (774/781)	76.7 (59.1–88.2) (23.30)	94.8 (93.1–96.2) (774/816)
	Culture and clinical data	40.0 (29.3–51.7) (28/70)	99.1 (98.2–99.6) (774/781)	80 (64.1–90.0) (28/35)	94.8 (93.1–96.2) (774/816)

**Table 4:** Performance of the direct 16S rRNA gene pan-mycobacterial PCR on pulmonary and extra-pulmonary specimens using clinical data to investigate discrepant results between smear microscopy, PCR and culture (culture and clinical data). Total of 952 samples. *PPV* positive predictive value, *NPV* negative predictive value

	Reference	Sensitivity % (95% CI)	Specificity % (95% CI)	PPV % (95% CI)	NPV % (95% CI)
Pulmonary (347)	Culture and clinical data	63.2 (52.7–72.6) (55/87)	99.2 (97.2–99.7) (258/260)	96.5 (88.1–99.4) (55/57)	89.0 (84.8–92.1) (258/347)
Pulmonary smear-positive (67)	Culture and clinical data	85.2 (73.4–92.3) (46/54)	100 (77.2–100) (13/13)	100 (92.3–100) (46/46)	61.9 (40.9–79.2) (13/21)
Pulmonary smear-negative (280)	Culture and clinical data	27.3 (15.1–44.2) (9/33)	99.2 (97.1–99.9) (245/247)	81.8 (52.3–96.8) (9/11)	91.2 (87.1–96.8) (245/269)
Extra-pulmonary (605)	Culture and clinical data	59.3 (46.6–70.9) (35/59)	99.1 (97.9–99.6) (541/546)	87.5 (73.9–94.5) (35/40)	95.7 (93.7–97.1) (541/565)
Extra-pulmonary smear-positive (34)	Culture and clinical data	72.7 (51.8–86.8) (16/22)	100 (75.7–100) (12/12)	100 (80.6–100) (16/16)	66.7 (43.7–83.7) (12/18)
Extra-pulmonary smear-negative (571)	Culture and clinical data	51.3 (35.9–66.5) (19/37)	99.1 (97.8–99.6) (529/534)	79.2 (59.5–90.8) (19/24)	96.7 (94.9–97.9) (534/571)

When considering only pulmonary specimens, the sensitivity, specificity, PPV and NPV were 63.2% (57/87), 99.2% (258/260), 96.5% (55/57) and 89.0% (258/347). When considering only smear-positive pulmonary specimens, the sensitivity, specificity, PPV and NPV were 85.2% (46/54), 100% (13/13), 100% (46/46) and 61.9% (13/21). When considering only smear-negative pulmonary specimens, the sensitivity, specificity, PPV and NPV were 27.3% (9/33), 99.2% (245/247), 81.8% (9/11) and 91.2% (245/269).

When considering extra-pulmonary specimens, the sensitivity, specificity, PPV and NPV were 59.3% (35/59), 99.1% (541/546), 87.5% (35/40) and 95.7% (541/565). When considering only smear-positive extra-pulmonary specimens, the sensitivity, specificity, PPV and NPV were 73.9% (16/22), 100% (12/12), 100% (16/16) and 66.7% (12/18). When considering smear-negative extra-pulmonary specimens, the sensitivity, specificity, PPV and NPV were 51.3% (19/37), 99.1% (529/534), 79.2% (19/24) and 96.7% (534/571).

**Fig. 3:**
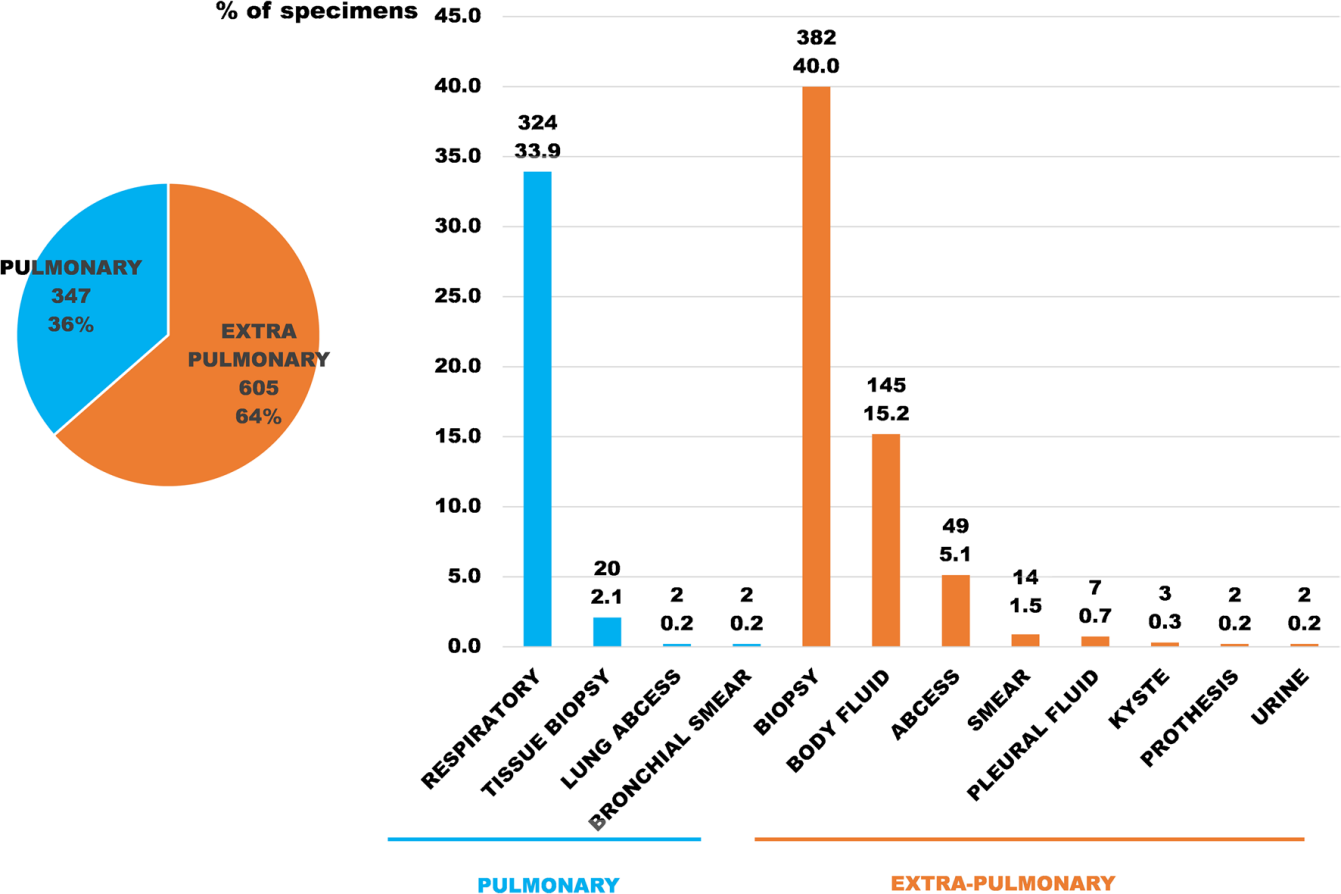
Specimens localisation (number of specimens and percentage)

These data suggest that the sensitivity and PPV of the pan-mycobacterial are satisfying on smear-positive specimens and limited on smear-negative specimens especially on pulmonary smear-negative specimens (Table 4 and Fig. 3).

## Comparison of the time to diagnosis of the pan-mycobacterial PCR and mycobacterial culture

We next addressed the potential added-value of the direct pan-mycobacterial PCR regarding the time to microbial diagnostic. The median time to microbiological diagnostic was 35 days when only culture was positive, while it was 6 days when the PCR was positive (*p* < 0.0001). When considering the subgroups of slow-growing mycobacteria, the time for identification was 35 days for culture and 6 days for the PCR (*p* < 0.0001), whereas for fast-growing mycobacteria, the time for identification was 26 days for culture and 6 days for PCR (*p* = 0.0001) (Table 5). When considering smear-positive and culture-positive specimens, the time for identification of culture and PCR was 29 days and 6 days, respectively (*p* < 0.0002), whereas considering only smear-negative and culture-positive specimens, the time for identification of culture and PCR was 35 days and 7 days respectively (*p* < 0.0001) (Table 5).

**Table 5:** Comparison of the time to diagnostic of the direct pan-mycobacterial PCR versus culture: (i) for fast- and slow-growing nontuberculous mycobacteria and (ii) according to the initial result of the smear examination. *nd* no data

	Number of patients	Median time to diagnostic (days)	Average time to diagnostic (days)	Standard deviation (days)
PCR-negative or not performed	164	35	38	18
Slow-growing NTM	145	35	39	18
Rapidly growing NTM	18	20	26	16
Mixed	1	85	85	nd
PCR-positive	30	6	7	3
Slow-growing NTM	21	6	7	3
Rapidly growing NTM	9	6	6	4
Total	194			
PCR-negative or not performed	164	35	38	18
Microscopy-negative	158	35	38	18
Microscopy-positive	6	30	37	28
PCR-positive	30	6	7	3
Microscopy-negative	9	7	7	4
Microscopy-positive	21	6	7	3
Total	194			

## Discussion

The microbiological diagnostic of nontuberculous mycobacterial infection has always been challenging because of the slow growth of these organisms. In addition, some mycobacterial species require specific growth conditions. Thus, despite a very low limit of detection (1–10 CFU per ml) culture may exhibit particularly limited sensitivity for the slowest growing mycobacteria, such as *M. genavense*, that may only be detected after 12-week incubation [13]. Moreover, culture do not apply for uncultivable organisms such as *M. leprae*. In the last decades, direct pan-mycobacterial PCR allowing mycobacteria detection and identification directly from clinical specimens have been introduced to circumvent these limitations. We aimed to evaluate the reliability and usefulness of the 16S rRNA gene pan-mycobacterial PCR that we use in routine in our diagnostic laboratory by comparing PCR with culture and microscopy during a 10-year period (2003–2013).

This study demonstrated that using a direct pan-mycobacterial PCR, the time to detection and identification of mycobacteria may be significantly reduced (29 days less) as compared with mycobacterial culture, which may represent a significant time saving for patient management. Despite exhibiting a very good specificity, the pan-mycobacterial PCR has a low sensitivity, even for smear-positive specimens (81.8%). This limited sensitivity of the broad-spectrum PCR is dependent on several factors as follows: (i) degenerated primers are used in order to extend the spectrum of the PCR; (ii) the length of the amplicon (approximately 800 base pairs) required to precisely assign the mycobacteria at species level; (iii) the amplicon detection methods, agarose gel chromatography, is less sensitive than real-time PCR detection with use of fluorescent probes; and (iv) the primers hybridise not only 16S rRNA gene from mycobacteria but also 16S rRNA gene from other *Actinobacteria* (i.e. *Corynebacterium* sp., *Nocardia* sp., *Actinomyces* sp. *Micrococcus* sp.), which is a problem for non-sterile specimens such as bronchial aspirates or skin. Relying on real-time PCR may improve the sensitivity of the method. Thus, a pan-mycobacterial PCR based upon a real-time PCR system will be developed ideally using both a highly conserved target such as the 16S rRNA gene to widely screen all mycobacteria and, in a duplex setting, using also a more discriminant target gene such as *rpoB* to allow precise identification of the most common nontuberculous mycobacteria, which mainly include *M. avium*, *M. kansasii* and *M. chelonae/abcessus* group [14]. Alternatively, multiplex real-time PCRs targeting the most common nontuberculous mycobacteria might be reliable [15–19]. Reaching 100% of sensitivity for smear-positive specimens, as for *M. tuberculosis* complex real-time PCR, would allow applying smear-independent algorithm for the diagnosis of NTM infections [6, 8]. Real-time PCR would also permit to circumvent the use of nested PCR, which is proposed by some to increase the sensitivity but which should be avoid whenever possible because of the high risk of specimen cross-contamination with amplicon; hence, we do not use nested-PCR anymore since several years in our molecular diagnostic laboratory [10, 20]. The negative predictive value reported in this study for the pan-mycobacterial PCR (93.3%) was high due to the low prevalence of NTM infections in this population. Specific mycobacterial culture thus remains necessary both for NTM detection and for subsequent phenotypic drug susceptibility test depending on the clinical situation. The time to identification at species level from positive culture might be improved by new approach such as identification using protein mass-spectrometry [21].

The time to result of the pan-mycobacterial PCR was not significantly impacted by the result of the smear examination. This might be due to the very low sensitivity of microscopy and the few number of smear-positive specimens. Similarly, the limited sensitivity of the pan-mycobacterial PCR on smear-positive specimens, might also be explained by the limited specificity of microscopy.

Our study identified 20 PCR-positive culture-negative specimens. Among these 20 specimens, 13 were considered true-positive based on another positive microbiological test (other specimen PCR positive or positive smear microscopy) or based on the clinical presentation of the patients and given the absence of documented PCR contamination. PCR-positive specimens with negative culture may also apply for (i) mycobacteria difficult to cultivate such as *M. genavense*, *M. marinum* or *M. ulcerans*; (ii) mycobacteria requiring specific growth conditions such as *M. haemophilum*; and (iii) mycobacteria impossible to cultivate in vivo, namely *M. leprae* [9, 11].

In conclusion, this study demonstrated that despite a yet limited sensitivity, the pan-mycobacterial PCR displayed an excellent specificity and significantly accelerated the time to diagnostic of NTM infections. Future developments should aim to introduce NTM real-time PCR with increased sensitivity in order to increase the detection rate and the negative predictive value. Such a sensitive PCR would permit considering a smear-independent algorithm of mycobacterial diagnosis to be quicker and more sensitive and less operator-dependent.

### Electronic supplementary material

ESM 1 (DOCX 20 kb)
