# Correction to: Added diagnostic value of 16S rRNA gene pan-mycobacterial PCR for nontuberculous mycobacterial infections: a 10-year retrospective study

**DOI:** 10.1007/s10096-024-04796-w

**Published:** 2024-03-27

**Authors:** Simon Andenmatten, Onya Opota, Jessica Mazza-Stalder, Laurent Nicod, Gilbert Greub, Katia Jaton

**Affiliations:** 1 Institute of Microbiology, University of Lausanne and Lausanne University Hospital, Lausanne, Switzerland; 2 Division of Pulmonology, University of Lausanne and University Hospital of Lausanne, Lausanne, Switzerland

**Correction to: European Journal of Clinical Microbiology & Infectious Diseases (2019) 38:1873–1881**

10.1007/s10096-019-03621-z

In the originally published version of this article, the names of the four authors were incorrectly presented as: Andenmatten Simon, Opota Onya, Greub Gilbert, Jaton Katia.

The correct names of the authors are: Simon Andenmatten, Onya Opota, Gilbert Greub, and Katia Jaton.

Also, the first name of the 3rd author was corrected from “Jesica” to “Jessica”.

The original article has been corrected.

