# Supplementary material for: Added diagnostic value of 16S rRNA gene pan-mycobacterial PCR for nontuberculous mycobacterial infections: a 10-year retrospective study
Source: Eur J Clin Microbiol Infect Dis. 2019 Jul 16;38(10):1873–81. doi: 10.1007/s10096-019-03621-z (PMC6778528; doi:10.1007/s10096-019-03621-z)
Supplement: Supplementary file 1 — (DOCX 20 kb) [file 10096_2019_3621_MOESM1_ESM.docx]

Added diagnostic value of 16S rRNA gene pan-mycobacterial PCR for nontuberculous mycobacteria infections: a 10-years retrospective study

Supplementary material

Table S1

| **Table S1** : Microbiological results and clinical data of patient’s specimens with positive pan-mycobacterial PCR and negative mycobacterial culture N = 19 | | | | | |
| --- | --- | --- | --- | --- | --- |
| **Sample** | **Specimen** | **Direct**  **examination** | **Results of the pan-mycobacterial PCR** | **Patient clinical presentation** | **Interpretation of the PCR result** |
| 1 | Liver | Positive | *M. genavense* | Disseminated disease, liver damages | Likely true positive |
| 2 | Bone | Positive | *M. genavense* | Disseminated disease | Likely true positive |
| 3 | Soft tissue | Positive | *M. marinum* or *ulcerans* | Lymphadenitis, granuloma | Likely true positive |
| 4 | Bone marrow | Positive | *M. genavense* | Disseminated disease/recurrence | Likely true positive |
| 5 | Sputum | Positive | *M. triplex* | Suspicion of tuberculosis | Likely true positive |
| 6 | Soft tissue, skin biopsy | Negative | *M. marinum* or *ulcerans (marinum)* | ulcer/Lymphadenitis | Likely true positive |
| 7 | Ganglion | Negative | *M. marinum* or *ulcerans (marinum)* | ulcer/Lymphadenitis | Likely true positive |
| 8 | Soft tissue | Negative | *Mycobacterium leprae* | Cutaneous lesion/hypoesthesia | Likely true positive |
| 9 | Bone marrow | Negative | *M. lentiflavum* (complex *M. simiae*) | Disseminated disease | Likely true positive |
| 10 | Bone | Negative | *M. genavense* | Disseminated disease/recurrence | Likely true positive |
| 11 | Central nervous system | Negative | *M. chelonae/abscessus* group | Abcess superinfection | Likely true positive |
| 12 | Prosthetic material | Negative | *M. smegmatis* | Prosthetic superinfection | Likely true positive |
| 13 | Liver | Negative | *M. chelonae/abscessus* group | Acute myeloid leukemia/hepatosplenic candidiasis | Unknown (may contaminant because of the Candida which might be the pathogen) |
| 14 | Ganglion | Negative | *M. chelonae/abscessus* group | Undetermined carcinoma w/t lymphadenopathy. | Unknown |
| 15 | Soft tissue | Negative | *M. avium* complex | Joint pain | Unknown |
| 16 | Abscess | Negative | *M. holsaticum,* *kyorinense* or *celatum* | Empyema | Unknown |
| 17 | Respiratory (Sputum) | Negative | *M. avium* complex | File not available | Unknown |
| 18 | Respiratory (Bronchial aspirate) | Negative | *M. parafortuitum* | File not available | Unknown |
| 19 | Soft tissue | Negative | *M. iranicum* | File not available | Unknown |
